# Clinical Potential of Regulatory T Cell Therapy in Liver Diseases: An Overview and Current Perspectives

**DOI:** 10.3389/fimmu.2016.00334

**Published:** 2016-09-06

**Authors:** Hannah C. Jeffery, Manjit Kaur Braitch, Solomon Brown, Ye Htun Oo

**Affiliations:** ^1^NIHR Biomedical Research Unit in Liver Diseases, Centre for Liver Research, Institute of Immunology and Immunotherapy, University of Birmingham, Birmingham, UK; ^2^Liver and Hepatobiliary Unit, University Hospital NHS Foundation Trust, Birmingham, UK

**Keywords:** regulatory T cells, microenvironment, metabolites, microbes

## Abstract

The increasing demand for liver transplantation and the decline in donor organs has highlighted the need for alternative novel therapies to prevent chronic active hepatitis, which eventually leads to liver cirrhosis and liver cancer. Liver histology of chronic hepatitis is composed of both effector and regulatory lymphocytes. The human liver contains different subsets of effector lymphocytes that are kept in check by a subpopulation of T cells known as Regulatory T cells (Treg). The balance of effector and regulatory lymphocytes generally determines the outcome of hepatic inflammation: resolution, fulminant hepatitis, or chronic active hepatitis. Thus, maintaining and adjusting this balance is crucial in immunological manipulation of liver diseases. One of the options to restore this balance is to enrich Treg in the liver disease patients. Advances in the knowledge of Treg biology and development of clinical grade isolation reagents, cell sorting equipment, and good manufacturing practice facilities have paved the way to apply Treg cells as a potential therapy to restore peripheral self-tolerance in autoimmune liver diseases (AILD), chronic rejection, and posttransplantation. Past and on-going studies have applied Treg in type-1 diabetes mellitus, systemic lupus erythematosus, graft versus host diseases, and solid organ transplantations. There have not been any new therapies for the AILD for more than three decades; thus, the clinical potential for the application of autologous Treg cell therapy to treat autoimmune liver disease is an attractive and novel option. However, it is fundamental to understand the deep immunology, genetic profiles, biology, homing behavior, and microenvironment of Treg before applying the cells to the patients.

Regulatory T cells (Treg) are critical regulators of immune tolerance (1). Regulatory activity among the CD25^+^ subclass of CD4^+^ T cells was first discovered in 1995 by Sakaguchi and colleagues *via* adoptive transfer studies. Depleting the CD25^+^CD4^+^ T cells from a T cell inoculum increased the rate at which graft versus host disease (GVHD) and features of autoimmune diseases developed in the recipient strain (2). The immunosuppressive potential of these cells was solidified in the result that replacement of the CD25^+^ fraction of CD4^+^ T cells could limit autoimmune disease induction (2, 3). CD4^+^CD25^+^ T cells constitute 5–10% of peripheral CD4 T cells in the blood, and they play a crucial role in maintaining immunologic self-tolerance by actively suppressing self-reactive lymphocytes (2). Treg development is controlled by FoxP3, which encodes the transcription factor that is genetically defective in an autoimmune and inflammatory syndrome in humans and mice (4, 5). IL-7 receptor, CD127 expression inversely correlates with FoxP3 and suppressive function of CD4^+^ Treg (6, 7); thus, Treg are currently defined as a subset of CD4 lymphocytes with the surface marker profile CD4^+^CD25^+^CD127^low^ and which express the intracellular transcription factor FoxP3. Treg are classified into two simple and broad categories; thymic-derived Treg (previously known as naturally occurring Treg) and peripheral Treg (previously labeled as adaptive Treg) (8).

## Profile of Regulatory T Cells in Liver Diseases

The majority of chronic active hepatitis is immune-mediated liver injury (9). Many investigators have reported Treg frequency variation in the peripheral blood in acute liver injury, chronic liver diseases, and liver cancer, but there are limited data on intrahepatic Treg. Reduction in CD4^+^CD25^high^CD127^low^ Treg frequency has been described in patients with alcoholic hepatitis (10). Progression from non-alcoholic fatty liver to non-alcoholic steatohepatitis is characterized by a higher frequency of Th17 cells in the liver and an increased ratio of Th17/resting CD4^+^CD45RA^+^CD25^high^ Treg in peripheral blood (11). We, and others, have reported that there is an increase in Treg frequency in parallel with effector immune cells in autoimmune liver diseases (AILD) (12–15). Treg also appear to play a role in the immunopathogenesis of primary biliary cholangitis (PBC) (16). Indeed, reduced FoxP3 expression in Treg has been described in the portal tracts of patients with PBC (17). Our group has previously reported the existence of a gut–liver link with the aberrant homing of mucosal T cells from the gut to the liver and extra-intestinal manifestations being seen in inflammatory bowel disease (18–20). Biliary epithelial inflammation has also been associated with the accumulation of CCR10-expressing Treg around the bile ducts in the liver (21).

In the setting of acute liver injury, such as acute viral hepatitis A, the size of the Treg pool was contracted due to Treg apoptosis *via* a Fas-mediated mechanism (22). Hepatitis B (HBV) pathogenesis is immunologically mediated and increased frequencies of CD4^+^ CD25^high^CD45RO^+^ Treg and cytotoxic T-lymphocyte-associated antigen 4 (CTLA-4) cells were noted in the peripheral blood of patients compared with controls and in patients who had recovered from a previous episode of HBV infection (23, 24). However, in HBV-related acute or chronic liver failure, while there was a reduction noted in CD4^+^ T cells, Treg numbers remained unchanged (25). In addition, serial biopsies from patients chronically infected with hepatitis C virus, taken during and after antiviral therapy, suggested that intrahepatic CD4^+^CD25^high^FOXP3^+^ Treg frequencies were increased upon interferon and ribavirin therapy in about half of patients, indicating stronger regulation of intrahepatic immunity by Treg during antiviral therapy (26). It is generally accepted that Treg are not beneficial in the setting of liver cancer as an increased Treg frequency correlates with CD8^+^ T cell impairment and poor survival of patients (27). All this body of evidence suggests that Treg play a major role in different types of liver diseases.

**Figure 1:**
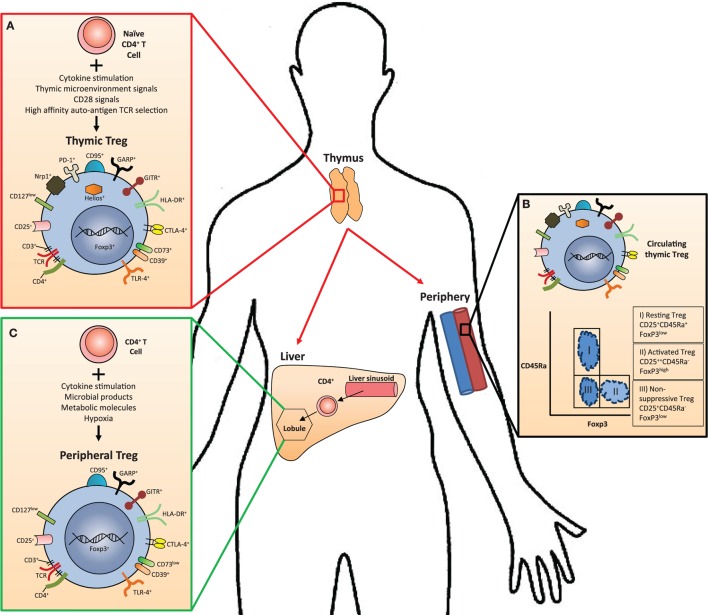
**The anatomical location of Treg cell subsets and their phenotypic markers in humans**. **(A)** Thymus-derived Treg (tTreg) cells are generated in the thymus from naive CD4^+^ T cells in combination with several stimulating factors. They express transcription factors and surface markers specific to all Treg subsets in addition to those specifically upregulated only in tTreg, such as PD-1, Helios, and Nrp-1. **(B)** Peripheral blood flow contains all circulating Treg subsets, including three subpopulations that may be defined by their expression of CD45Ra and FoxP3. **(C)** In tissues such as the liver, naive CD4^+^ cells may differentiate into peripherally derived Treg cells (pTreg) in response to stimulating factors. These cells express many of the same transcription factors and surface markers as tTreg with notable differences, such as reduced Helios, Nrp1, CD73, and PD-1 expression.

The Treg population has been classified as CD25^+^CD45RA^+^FOXP3^low^ resting, CD25^++^CD45RA^neg^FOXP3^high^ activated, suppressive Treg and CD25^+^CD45RA^neg^FOXP3^low^ cytokine-secreting non-suppressive Treg (28) (Figure 1). We recently described intrahepatic Treg as predominantly effector memory lymphocytes with PD1^low^, CD69^high^ phenotype (15) and expressing both hepatic homing CXCR3 and biliary tropic CCR6 chemokine receptors (14) (Figure 1).

## Suppressive Mechanisms of Regulatory T Cells

**Figure 2:**
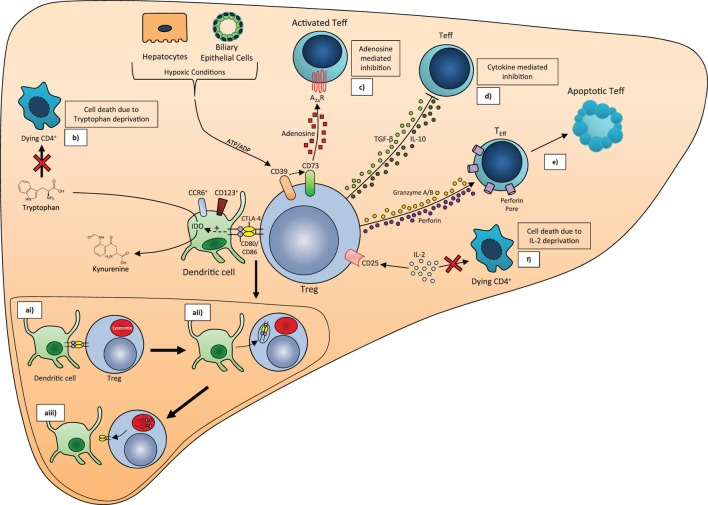
**Treg mechanisms of action**. (a) CTLA-4/CD80/CD86 trans-endocytosis. (ai) CTLA-4 on the surface of Treg binds to CD80/CD86 on the dendritic cell surface. (aii) CTLA-4 and CD80/CD86 are brought into the Treg *via* endocytosis. (aiii) CD80/CD86 fuses with lysosomes to be broken down, while CTLA-4 is recycled to the cell membrane. (b) IDO-mediated tryptophan deprivation. Binding of CTLA-4 and CD80/CD86 stimulates induction of the enzyme Indoleamine 2, 3-dioxygenase (IDO) in CD123^+^CCR6^+^ dendritic cells, which catalyzes the conversion of Tryptophan to N′-Formylkynurenine. The resulting Tryptophan depletion leads to CD4^+^ cell death. (c) Conversion of ATP to adenosine *via* CD39/CD73. CD39 and CD73 expressed on the Treg cell surface convert ATP/ADP released from respiring hypoxic cells into adenosine, which binds to receptors on activated T effector (Teff) with an inhibitory effect. Depletion of ATP also suppresses Teff proliferation. (d) Cytokine release. TGF-β and IL-10 released by Treg inhibit Teff cell proliferation and activation. (e) Induction of apoptosis. Release of Granzyme A, Granzyme B, and perforin by Treg leads to apoptosis of Teff. (f) IL-2 deprivation. Deprivation of IL-2 from CD4^+^ T cells by CD25 on the Treg surface leads to cell death.

Regulatory T cells are potent mediators of self-tolerance in the periphery and function *via* multiple mechanisms to achieve immune modulation. Treg exert their functions by (i) inhibiting the function or maturation of antigen-presenting cells (APCs), (ii) destroying target cells by inducing apoptosis, (iii) causing metabolic disruption *via* the adenosine pathway, and (iv) by secreting immunosuppressive cytokines, transforming growth factor beta (TGF-β), and IL-10 or competitive consumption of survival cytokines in particular IL-2 (Figure 2).

T-lymphocyte-associated antigen 4 (CTLA-4) protein expression on Treg plays an important role in the suppressor function of Treg (29). In particular, deficiency of CTLA-4 in Treg impairs their suppressive function to result in fatal T cell-mediated autoimmune disease. CTLA-4 *via* the process of trans-endocytosis depletes its two ligands, CD80 and CD86 from dendritic cells (DCs), thereby removing their availability to act as costimulatory ligands through CD28 (30) (Figure 2). *In vitro* studies have also identified that CTLA-4 can additionally suppress T-cell proliferation *via* upregulation of several essential amino acid consuming enzymes, including indolamine 2,3-dioxygenase, histidine ammonia lyase, nitric oxide synthase 2, and l-threonine 3-dehydrogenase in APC through binding CD80 on the APC cell surface (31). IDO-positive DCs represent a regulatory subset of APCs in humans (32) (Figure 2). Regulation of tryptophan metabolism by indolamine 2,3-dioxygenase (IDO) in DCs is a highly versatile modulator of immunity. IDO converts tryptophan to kynurenine, which inhibits the proliferation of effector T cells (33) (Figure 2) but promotes FoxP3 induction in a mechanism involving reduced PI3K/mTOR signaling (31). Importantly, we have shown that human intrahepatic Treg reside close to DCs and effector T cells to exert their suppressive function in the areas of chronic hepatitis, either in the lobules or in the areas of interface hepatitis (34).

**Figure 3:**
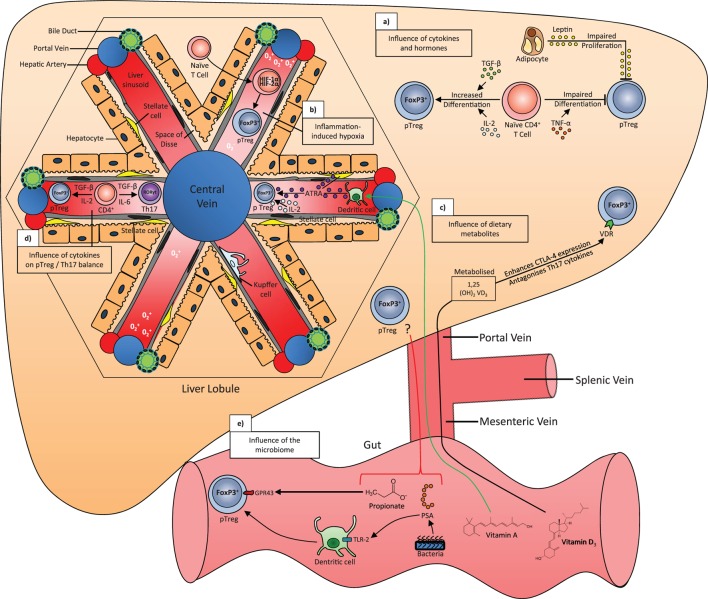
**The influence of cytokines, hypoxia, dietary metabolites, microbiota, and hormones on the generation of peripherally derived regulatory T cells (pTreg) in the liver**. (a) TGF-β and IL-2 signal to promote the differentiation of naive CD4^+^ T cells, while TNF-α impairs differentiation. Leptin released by adipocytes impairs the proliferation of pTreg cells. (b) Cytokines released during liver inflammation can lead to hypoxic conditions, which stabilizes the transcription factors hypoxia-inducible factor 1α and 2α (HIF-1α/HIF-2α) in naive CD4^+^ T cells. These factors stimulate FoxP3 expression and a move toward a pTreg phenotype. (c) Dietary vitamin D3 brought to the liver *via* the portal vein is metabolized into its active form 1,25 (OH_2_) VD3 or calcitriol, which enhances CTLA-4 expression and antagonizes Th17 cytokines in all T cells, including pTreg, *via* the vitamin D receptor (VDR). Dietary vitamin A is converted to all-trans retinoic acid (ATRA) by CD103^+^ dendritic cells in the liver sinusoid, which in combination with ATRA released by stellate cells and IL-2, stabilizes FoxP3 and the pTreg phenotype. (d) TGF-β in combination with IL-2 or IL-6 will differentiate naive CD4^+^ T cells into pTreg or Th17 cells, respectively. (e) Propionate is a short-chain fatty acid that is metabolized by gut microbes and binds to GPR43 receptor to stabilize the pTreg phenotype. The bacteria component polysaccharide A (PSA) binds to TLR-2 on dendritic cells, which increases pTreg differentiation. The influence of these products in the liver is unknown.

TGF-β1, an immunosuppressive cytokine, supports the maintenance of FoxP3 expression, regulatory function, and homeostasis in peripheral Treg (35). It is also a crucial cytokine, which dictates the development of a Treg versus Th17 lineage (36) and limits T effector proliferation *via* induction of essential amino acid catabolizing enzymes in APCs. IL-10 released by Treg also promotes essential amino acid depletion *via* upregulation of certain catabolizing enzymes in APCs (31). The liver is enriched with TGF-β1 (37, 38) (Figure 3). Recently, CD103^+^ intestinal DCs have been shown to promote a tolerogenic environment *via* integrin αvβ8-mediated activation of TGF-β (39), and DCs lacking αvβ8 fail to induce Treg (40).

Treg could also suppress or kill responder T cells after cell to cell contact in a perforin-dependent and independent manner by granzymes A and B (41, 42). Treg can also inhibit T cell proliferation by inducing pericellular adenosine from extracellular nucleotides (43, 44), which is catalyzed by ectoenzymes, CD39, and CD73 expressed on Treg (45, 46). The coordinated expression of CD39/CD73 on Treg and the adenosine A2A receptor on activated T effector cells generates immunosuppressive loops, implementing the inhibitory function of Treg cells (43, 47) (Figure 2). We reported previously that there is an increase in the frequency of intrahepatic Treg in inflamed human livers (34) in parallel with total CD4 T cells, and they had high expression of their functional markers such as CTLA-4, CD39, and also secreted IL-10.

IL-2 is a potent inducer of T-cell proliferation and T-helper cell differentiation and is necessary for the survival and function of memory T cells. It is also important for the development, survival, and function of Treg (45, 48, 49). Treg have higher expression of the high-affinity IL-2 receptor subunit, CD25, than effector T cells; thus, Treg can suppress effector cell proliferation and differentiation by competitive consumption of IL-2 when located in close proximity (50). However, the functional capacity of intrahepatic Treg is reduced in the IL-2-deficient inflamed hepatic microenvironment (15). We demonstrated that the main source of intrahepatic IL-2 is activated T effector cells, and this cytokine is crucial for Stat5 signaling in Treg (15). IL-2 is also required for the homeostatic maintenance of Treg. Taken together, such low intrahepatic IL-2, which appears reduced further in disease, might contribute to a lack of Treg-conferred protection and the progression of immune-mediated liver diseases. Indeed, autoimmune diseases can be induced by IL-2 neutralization or defective IL-2 production (51, 52).

Thus, Treg exert suppression in multiple pathways. The mechanism is dependent on the specific tissue and its microenvironment (Figure 2).

## Hepatic Microenvironment and Regulatory T Cells

Intrahepatic Treg are residing in a microenvironment, which is deprived of oxygen and enriched with microbes, metabolic products; inflammatory cytokines, and hormones, which all play a role in Treg differentiation and function (Figure 3). The liver is enriched with fat-soluble vitamins. All-Trans retinoic acid (ATRA), a metabolite of vitamin A, is enriched in Ito cells (stellate cells) and plays a pivotal role in maintaining the stability and function of Treg in the inflammatory milieu (53, 54). Retinoic acid can also directly promote TGF-β-mediated conversion of naive T cells to cells of FoxP3^+^ Treg phenotype (55, 56). In addition, retinoic acid can prime human DCs to induce gut homing, IL-10-producing Treg (57) (Figure 3).

The active form of vitamin D, 1,25-dihydroxyvitamin D3 and IL-2 synergistically combine to inhibit T cell production of inflammatory cytokines and promote development of Treg expressing CTLA-4 and FoxP3 (58) (Figure 3). One recent report showed that short-chain fatty acids (SCFA) from gut microbiota-derived bacterial fermentation products regulate the size and function of the colonic Treg pool and protect against colitis, suggesting that abundant microbial metabolites underlie adaptive immune microbiota coadaptation and promote colonic homeostasis (59). We have also reported that the inflamed liver microenvironment is enriched with pro-inflammatory cytokines IL-1, IL-12, IL-6, IL-8, and TNFα, but deprived of the Treg survival cytokine IL-2 (15). Nonetheless, although they present a somewhat reduced regulatory capacity, intrahepatic Treg still possess an intact functional capacity and maintain their lineage for a short time in culture conditions that mimic the intrahepatic environment (15).

There is a shift in metabolic supply-and-demand ratios during inflammation. Tissue hypoxia within inflammatory lesions dictates an anti-inflammatory program by driving expression of hypoxia-inducible factor (HIF)-1α that acts to increase the frequency and suppressive properties of thymic Treg (60, 61). Indeed, hypoxic Treg were more effective than normoxic cells in suppressing the proliferation of effector lymphocytes (61). The hepatic microenvironment is a hypoxic atmosphere especially around zone 3, where cells are closer to the central vein and most distant from the hepatic artery oxygen supply. Thus, while not proven, a gradient in Treg potency might be expected through the liver.

The liver is continuously exposed to gut microbes and bacterial toxins *via* its portal venous flow. It is clear that the microbiome has a strong influence on the immune system. For example, breast-fed infants develop robust populations of memory T cells as well as T helper 17 (Th17) cells within the memory pool, whereas bottle-fed infants do not, and this may partly explain the variation in human susceptibility to conditions with an immune basis, as well as the variable protection against certain infectious diseases (62). Oral bacteria administration in mice also promotes Treg and alleviates bowel inflammation in a model of immune-mediated colitis (63); thus, the microbiome may serve as a target for future Treg-based immunotherapies.

## Recruitment and Positioning of Treg to the Inflamed Liver

Leukocyte trafficking and positioning within tissues is directed by chemokines. Thus, chemokines play critical roles in regulating immune responses and inflammation (64, 65). Chemokines can be classified into “inflammatory” and “homeostatic/constitutive” based on whether they are induced by inflammation or constitutively expressed and involved in homeostatic immune regulation (64). Hepatic Treg express a unique range of chemokine receptors, which interact with corresponding chemokines, and these receptors are crucial for their homing and positioning in the inflammatory liver tissues. Human intrahepatic Treg express the chemokine receptor CXCR3 for recruitment across hepatic sinusoids, CCR4 for positioning close to hepatic DCs and both CXCR3 and CCR6 chemokine receptors for positioning around bile ducts (15, 34). This chemokine receptor expression profile is essential for Treg to locate at the site of inflammation and to interact with other immune cells. CXCR3 deficiency has been shown to exacerbate liver disease and abrogates tolerance in mouse models of immune-mediated hepatitis (66).

## Clinical Application of Treg

As has been discussed, Treg play a pivotal role in controlling the magnitude of immune responses to provide tolerance to self-antigens and to limit tissue damage caused by immune activation in response to innocuous antigens. Given the contribution of aberrant immune control in the progression of disease including: (1) suppressed effector immune response which results in unwanted Treg activity in cancer, (2) impaired immune-regulatory function in autoimmune or inflammatory diseases, and (3) the deleterious consequences of life-long immunosuppression therapy following organ transplantation, the prospect to control disease progression through targeting the regulatory cells in settings of cancer, solid organ and hematopoietic cell transplantation, transplant rejection, and autoimmune diseases has been an attractive option for clinicians over many years.

The cytokine IL-2 is essential for the function and expansion of Treg (49). Thus, to improve immune regulation through low-dose, IL-2 therapy, which targets the Treg selectively in contrast to effector, has been tested with positive outcomes in human autoimmune-related diseases in Phase I and II settings (67–70).

The establishment over recent years of good manufacturing practice (GMP)-compliant reagents and equipment that can allow the isolation of cells according to their expression profile of certain surface proteins has made it possible to isolate Treg from the peripheral circulation as a cell immunotherapy. The concept underlying this “*Regulatory T cell therapy*” is that administering a concentrated source of a desired population of Treg can tip the balance of the patient’s immune system to enhance its regulatory capacity. It builds on the knowledge that a lack of Treg function due to mutation of the Treg lineage-defining transcription factor FOXP3 leads to the X-linked autoimmune syndrome immune dysregulation, polyendocrinopathy, enteropathy X-linked syndrome (IPEX) (71), and, upon the seminal observation by Sakaguchi and colleagues, that giving CD4^+^CD25^+^ Treg could prevent autoimmune disease (2). In this section, we discuss developments in the field of Treg immunotherapy and its potential to be used to treat liver diseases in the future.

## Generation of GMP-compliant Clinical Grade Treg for Cell Therapy

To be used therapeutically, a cellular product must be deemed GMP-compliant. This has massive implications upon its production compared with a basic laboratory reagent as the final product and all the reagents and equipment used in its manufacture must exceed a standard clinical quality (purity) and sterility. Accordingly, all staff involved in its preparation must be highly skilled in aseptic procedures and must receive regular re-training. There must be a detailed log of the production and characterization of the final product. Altogether, this imposes extremely high overheads to the production of cellular therapies and with regard to Treg cell therapy has further limited the precise phenotypic character that can be achieved both for practical and for financial reasons. Consequently, the Treg used in trials until now have differed.

**Figure 4:**
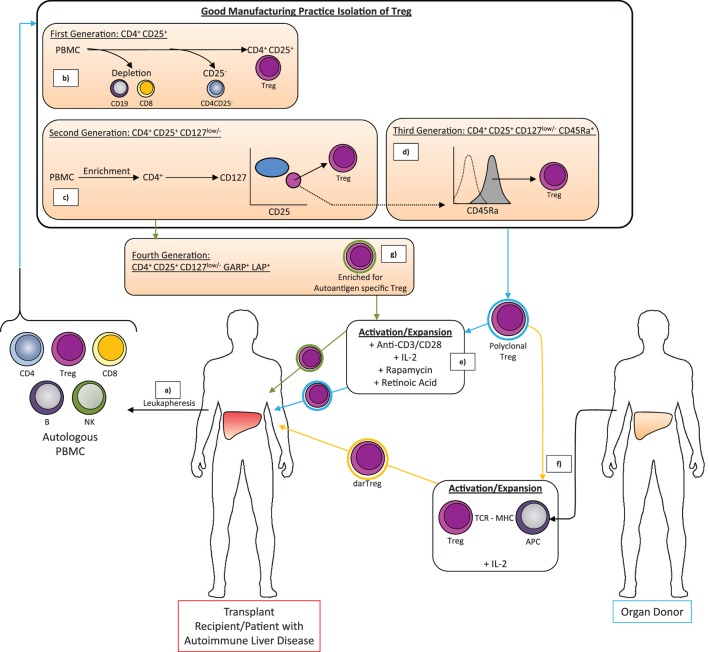
**The good manufacturing practice-compliant methods for isolation and expansion of Treg for therapeutic application in liver transplantation or treatment of autoimmune liver diseases**. (a) Peripheral blood mononuclear cells (PBMC) are isolated from the peripheral blood of the patient by density gradient centrifugation of the leukapheresis product. (b–d) Different approaches may then be used to isolate polyclonal Treg characterized by certain combinations of surface markers. The absolute phenotype of Treg that can be purified depends on the availability of GMP-compliant antibody-coated microbeads and/or flow cytometry antibodies as well as a GMP magnetic sorting system and/or GMP flow sorting facility. (b) CD4^+^CD25^+^ Treg are isolated by microbead depletion of CD8^+^ T cells and CD19^+^ B cells followed by microbead positive isolation of CD25^high^ cells. Short incubation at low temperature with anti-CD25 microbeads helps recover only those cells expressing CD25 at high levels (CD4^+^ Treg). (c) Microbead-based enrichment of CD4^+^ T cells followed by flow sorting for CD25^high^ CD127^low/−^ cells allows isolation of the bona fide (second generation) Treg population with greater percentage FoxP3^+^ Treg than first generation Treg. (d) Additional selection based on CD45Ra expression by flow sorting can isolate a Treg population with enhanced propensity to proliferate. (e) The isolated Treg are a polyclonal population (blue halo) but the product is insufficient in number for therapeutic efficacy. (e) Treg are, therefore, expanded by culture with anti-CD3/anti-CD28-coated expansion beads in the presence of IL-2 to promote proliferation and survival, rapamycin to kill contaminating T effector cells and retinoic acid to enhance the Treg phenotype. These polyclonal-expanded autologous Treg may then be infused to the patient. However, donor alloantigen-reactive Treg (darTreg) (orange halo) have greater potency in preventing graft rejection following transplantation and can be isolated from the initial polyclonal Treg pool by culture with antigen-presenting cells from the donor (f). Similarly, against autoimmune disease, autoantigen-specific Treg are expected to have greater potency (green halo). In many cases, the offending autoantigen is not known but isolation of Treg based on markers such as latency-associated peptide (LAP) and glycoprotein A repetitions predominant (GARP) might help to generate a population that has enrichment of autoantigen-specific Treg and which following non-specific expansion with anti-CD3/anti-CD28-coated microbeads can be returned to the patient to provide stronger regulation of inflammation than a polyclonal preparation (g).

To date, we can define three categories of GMP-grade clinical Treg: first generation (CD4^+^CD25^+^); second generation, bone fide Treg (CD4^+^CD25^+^CD127^low/−^), and third generation naive Treg (CD4^+^CD25^+^CD127^low/−^CD45Ra^+^) (Figure 4). There are two options to achieve GMP clinical grade Treg, bead-based GMP technology and flow sorting technology. While first generation Treg can be isolated by magnetic bead-based approaches alone, such as CD8 and CD19 depletion followed by CD25 enrichment, there are no GMP bead reagents to deplete CD127-expressing effectors. Isolation of second and third generation Treg that are exclusively CD127^low/−^ depends on the availability of a GMP-compatible flow sorting facility. Tyto technology (Miltenyi Biotec) now exists that can facilitate the isolation and expansion of a highly pure second (CD4^+^CD25^+^CD127^low/−^) and third generation (CD4^+^CD25^+^CD127^low/−^CD45Ra^+^) GMP Treg preparation straight from a leukapheresis product within a closed system, reducing the risk of loss of sterility and optimizing the purity of the preparation from the start. Moreover, by including Rapamycin and Retinoic acid in the culture cocktail during subsequent expansion, it is possible to ensure that loss of purity through the outgrowth of any contaminating effector T cells is not of concern. Rapamycin precludes the expansion of effector but not regulatory T cells *via* differential downstream signaling from the IL-2R (72). Furthermore, additional supplementation with IL-2 ± retinoic acid can help to support Treg proliferation and potentiate the functional phenotype (73), and it is possible to measure the demethylation status of the Treg-specific demethylated region (TSDR) prior to administration of the cell therapy product to the patient to verify purity. Although percentage demethylation boundaries are not yet rigorously defined, such could be implemented during trials to help restrict any adverse events based on known lack of purity.

## Treg Immunotherapy for Liver Transplantation and Autoimmune Liver Diseases

To date, GMP Treg therapy has not been reported in the treatment of the liver diseases either in preventing rejection of donor organs, preventing GVHD posttransplant, enabling withdrawal of immunosuppressants or overcoming any need for them posttransplant, or in inducing or maintaining states of remission in the pre-transplant setting, but evidence from animal studies (2, 3, 74, 75) and trials of Treg immunotherapy in other human disease backgrounds provide sound rationale for the attempted use of this therapy in liver medicine in the future. This is particularly important in an era when the demand for liver transplants exceeds the availability of matched donor livers. Currently, the first clinical trials are underway, investigating the safety and feasibility of Treg therapy in renal transplantation (76, 77), and it is likely that outcomes of this trial could be applicable to similar solid organs such as the liver.

### Application of Treg Immunotherapy to Induce Liver Transplantation Tolerance

Successful solid organ transplantation requires the regulation of two immune reactions: (i) antigenic activation of the donor immune system against the host that if not restrained leads to GVHD and (ii) antigenic activation of the host immune system toward the graft, which can lead to graft rejection. GVHD is a major complication in the setting of bone marrow transplantation, owing to the preponderance of T cells in bone marrow, but in solid organ transplant it is generally of lesser concern. Moreover, the tolerogenic nature of the liver and its ability to regenerate (78) means that the recipient–donor match in liver transplant need be less stringent and the recipient to donor immune response is perhaps the area of gravest concern therapeutically. This is currently targeted through life-long immunosuppression, but leaves individuals at risk of opportunistic infections, diabetes, hypertension, and malignancy (79).

The first human studies of Treg cell therapy explored its potential in the regulation of GVHD and tested HLA-matched sibling donor-derived expanded CD4^+^CD25^+^CD127^low/−^ (second generation) Treg to combat the difficulty in treating GVHD, following allogeneic bone marrow or stem cell transplantation (80, 81). Although, these initial studies had mixed outcomes, they provided hope for Treg therapy in GVHD and highlighted the possible importance of timing and dose. A more recent Phase I dose escalation study of Treg tested the use of partially HLA-matched donor umbilical cord blood as opposed to peripheral blood (82).

Excitingly, Todo and colleagues brought a new concept of Treg-based therapy to the table of operational tolerance in living donor liver transplantation (83). In this instance, the straightforward coculture of irradiated donor lymphocytes collected from a leukapheresis product with recipient lymphocytes in the presence of anti CD80/86 monoclonal antibodies generated a recipient cell product that proved enriched with cells of regulatory phenotype. *In vitro*, these cells inhibited the proliferation of recipient cells in response to donor cell stimulation in a dose-dependent manner, and, *in vivo*, the therapy allowed immunosuppressive agents to be tapered and completely discontinued within 18 months. This is revolutionary in the field as at the time of reporting 7/10 subjects had had successful weaning and remained drug free for up to 33 months. Successful weaning from immunosuppressant has previously been described between 3 and 11 years following transplant with longer delay giving higher (up to 80%) success (84). Only three individuals who were patients with autoimmune liver disease did not tolerate the novel therapy well, and, thus, its potential in the setting of autoimmune liver disease transplantation tolerance requires further consideration (83).

### Application of Treg Cell Therapy to Restore Peripheral Immune Tolerance in Autoimmune Liver Diseases

Regulatory T cell therapy has not yet been tested in the AILD in humans. Since there have been no advances in new therapies for these diseases in the last three decades, observations of reduction of Treg and/or impairment of Treg function in these diseases supports the concept of Treg therapy in AILD (12, 15, 17, 85). Treg therapy is an attractive option and especially now that standard operating procedures (SOPs) and GMP reagents and equipment are in place for the automated and safe manufacture of patient-specific cell-based therapies. It is also being considered as a future therapy for a number of other autoimmune diseases, including inflammatory bowel disease and Crohn’s disease (86, 87), rheumatoid arthritis (88), and systemic lupus erythematosus (SLE) (89). Although a lack of regulatory control is clear in these diseases, the precise roles of Treg in the pathogenesis of these diseases are unclear as studies have been contradictory with regard to changes in frequency and impaired functionality, and it may be that the lack of control is due to defects in the capacity of the antigen-presenting and/or effector cells to be downregulated by the regulatory cells.

To date, the seminal pioneering studies in this arena have been in type-1 diabetes, an autoimmune disease of the pancreatic beta islet cells wherein destruction of the beta islets by autoreactive T cells leads to loss of production of the hormone insulin, which is vital to the regulation of blood glucose. The first safety study in humans was in 2012 in a pediatric cohort. Ten children were given 1 or 2 × 10^7^ CD4^+^CD25^+^CD127^low/−^Treg/kg body weight without any adverse events. Significant increases in the proportions of peripheral Treg were measured after transfer and importantly at 6 months post treatment. Eight out of the 10 children, receiving Treg therapy, were in remission with 2 weaned off from insulin therapy completely. This was in comparison to the control cohort, all of whom continued to require insulin therapy (90).

Subsequent work by Tang and colleagues has evaluated safety in adults, testing escalating doses of CD4^+^CD25^+^CD127^low/−^ Treg between 5 × 10^6^ and 3.2 × 10^8^ cells. No serious adverse events were observed with the dose escalation study, and an improvement in C-peptide level was noted (91). By including [6,6-^2^H_2_] glucose, which incorporates into the deoxyribose moiety of newly synthesized DNA during expansion, the researchers were able to non-radioactively label the Treg and follow their persistence in the patient following infusion. Excitingly, although only 25% of the peak label was retained on cells at 90 days, it could still be detected up to 1 year, suggesting maintenance of the infused Treg (91). Furthermore, expressions of Treg-defining markers, including FoxP3, CD25, and CD127, remained stable in Treg post infusion, and there was not a shift toward a T effector cytokine profile. Thus, these studies in type-1 autoimmune diabetes provide good support for attempting Treg immunotherapy to treat AILDs, including autoimmune hepatitis (AIH), primary sclerosing cholangitis (PSC), and PBC in the future. However, no study has yet verified that Treg cells administered actually reach the site of disease, establish persistence at the site and maintain a stable phenotype overtime. The recent study by Todo and colleagues applying Treg in the setting of living donor liver transplant did perform liver biopsies at regular intervals during follow up to detect the graft inflammation and fibrosis, but no immunology study was done on the liver allograft after Treg infusion to investigate the localization and fate of the infused cells at the tissue level (83). To fully understand outcomes of therapeutic efficacy in future trials of the therapy, it would be of advantage if these questions could be tackled through biopsy and or/novel *in vivo* imaging methods post treatment.

## Optimization of Regulatory T Cell Therapy for Application in the Liver Diseases

Taken together, studies using GMP Treg, whether first or second generation, appear promising although clearly the optimization of factors including dose, timing relative to transplant/disease stage, as well as the choice of immunosuppression regimen and the frequency of Treg reapplication are important, and it is likely that each of these will be dictated by the individual situation (prophylactic pre or posttransplant versus autoimmunity/GVHD/rejection), the grade of the individual’s disease, and whether the patient’s disease is in remission (requiring withdrawal of standard medication) or in relapse (requiring settling of active immune reactions as well as withdrawal of standard medications). Given that Cyclosporine reduces Treg number, immunosuppression therapies used together with Treg therapy should be tailored to a rapamycin-based regimen along with Tacrolimus, which increases Treg survival. Patients with active viral infection and previous history of malignancy should be excluded from any Treg clinical trial as Treg would have a negative impact on the disease process.

It seems likely that isolated cells will have to be expanded in order to be of adequate number to be used in therapy. Hence, one caveat will, therefore, be the reliable expansion of autologous Treg cells for all patients since most studies, to date, have reported a few subjects who were enrolled but failed to receive treatment or treatment at the intended dose due to an inadequate cell yield.

The question over the preparation of the Treg also remains. Until now the Treg tested in clinical trials have been polyclonal and, thus, have ability to function through indirect pathways but also specifically due to a number within the mix that have donor antigen specificity (namely alloantigen-specific Treg). With a view to optimize Treg for transplantation therapy, polyclonal and alloantigen-specific Treg have been compared. Alloantigen-specific Treg represent those Treg that are activated by donor-specific antigens and can be expanded from polyclonal Treg populations in mixed lymphocyte reactions with donor APCs. By contrast polyclonal Treg are expanded with anti-CD3/anti-CD28-coated expander beads. Alloantigen-specific T cells can be identified and selected for further expansion based on their induction of activation markers such as CD69 and CD71. The fact that the therapeutic product created by Todo et al. through mixed lymphocyte reaction had greater suppressive ability over recipient T cells activated by donor as opposed to third-party stimulation was clear evidence of underlying allospecific regulatory activity having been raised within the expanded culture, and, as such, using a system to generate greater donor specificity might be advantageous and should allow considerably reduced numbers of Treg to be used (83). The value of alloantigen-specific Treg in transplantation tolerance was cleverly demonstrated *in vivo* using a humanized mouse model of human skin graft immune rejection. Allogeneic Treg expanded on myeloid DCs from the skin were more effective at limiting dermal and inflammatory phenotypes of skin rejection by infusions of allogeneic T effector cells than polyclonal Treg (74). Various methods have been proposed to generate GMP-compliant donor alloantigen reactive Treg (darTreg) for therapeutic application. Appropriate alternative cell types to incorporate in coculture with recipient CD4^+^CD25^+^ Treg to drive the expansion of darTreg include donor PBMCs, donor monocyte derived DCs, and donor B cells (92) activated on 3T3 fibroblasts expressing CD40L (93). A number of current phase 1 and phase 2 clinical trials propose the application of darTreg as opposed to polyclonal Treg (Table 1). Similarly, in autoimmunity, where models of disease propose the likely hood of only a defined autoantigen, antigen-specific Treg may be the way forward, but this requires knowledge of the offending antigen. In the liver diseases, autoantigens for type II AIH (94) and PBC (95) are known but for type I AIH and PSC are yet to be discovered precluding the benefits of antigen-specific therapy at the present time. Nonetheless isolation of Treg expressing markers of activation such as latency-associated peptide (LAP) and glycoprotein A repetitions predominant (GARP) might help to generate a product with a higher proportion of antigen-specific Treg (Figure 4).

**Table 1:** **Clinical trials in regulatory T cell therapy in solid organ transplantation or autoimmune diseases that were listed as recruiting in the www.ClinicalTrials.gov registry at the time of manuscript preparation (accessed 04-05-2016)**.

Title of study/www.ClinicalTrials.gov identifier	Sponsor	Location of study	Start date/end date/enrollment/phase	Purpose of the study
*deLTa*: darTregs in liver transplantation/NCT02188719	NIAID	1. University of California San Francisco, USA	December 2014/January 2022/24/phase 1	To evaluate the safety of taking a specific combination of immunosuppressant drugs after liver transplantation and the safety of receiving one of three doses of darTregs while taking this combination of immunosuppressant drugs
		2. Mayo Clinic Minnesota, USA		
*The ONE study*: Infusion of Treg in kidney transplant recipients/NCT02091232; The ONE study is a unified approach to evaluating cellular immunotherapy in solid organ transplantation	Massachusetts General Hospital	Collaboration of US and EU Centers	May 2014/May 2018/8/phase 1	To test different types of Treg for safety and the promotion of kidney survival
				To examine in living donor renal transplant recipients the safety and feasibility of administering Treg derived from recipient PBMC stimulated with kidney donor PBMC in the presence of costimulatory blockade with belatacep
*ONETreg1*: The ONE study UK Treg Trial/NCT02129881.	Guy’s and St Thomas’ NHS Foundation Trust	1. Guy’s Hospital, London, UK	April 2014/March 2017/12/phase 1 and phase 2	To asses autologous expanded polyclonal Treg as a treatment to prevent kidney transplant rejection with infusion into the patient 5 days after kidney transplant for end-stage renal failure
		2. The Oxford Transplant Centre, Oxford, UK		
*DART*: darTreg therapy in renal transplantation. The ONE study US Treg Trial/NCT02244801	University of California, San Francisco	University of California, San Francisco	November 2014/June 2018/16/phase 1	To evaluate the safety and tolerability of darTreg infusion for adult, *de novo* living donor renal transplant recipients
*ARTEMIS*: darTregs for calcineurin inhibitor reduction/NCT02474199	NIAID	1. University of California at San Francisco, USA	September 2015/December 2018/18/Phase 1 and phase 2	To examine the safety of one dose of darTreg and to see if the Treg allow the recipient of a living donor liver transplant to take less or completely stop the medications normally taken after receiving an organ transplant
		2. Mayo Clinic, Minnesota, USA		
*ThRIL*: Safety and efficacy study of Treg therapy in liver transplant patients/NCT02166177	Guys and St Thomas’ NHS Foundation Trust	King’s College Hospital	June 2014/June 2019/26/phase 1 and phase 2	To examine the feasibility, safety and efficacy of an autologous Treg product as an adjunct immunosuppressive treatment in liver transplantation
*TASK*: Treg adoptive therapy for subclinical inflammation in kidney transplantation/NCT02088931	University of California, San Francisco	University of California, San Francisco	March 2014/February 2016/3	Pilot study of CD4^+^CD127^low/−^CD25^+^ polyclonal Treg adoptive immunotherapy in renal transplant recipients. The aim is to test the safety of a single infusion of autologous expanded Treg
*TASK*: Treg therapy in subclinical inflammation in kidney transplantation NCT02711826	NIAD	University of California, San Francisco	May 2016/April 2018/45	To see if polyclonal Treg or darTreg can reduce inflammation in a transplanted kidney and find out the effects of taking everolimus after polyclonal Treg or darTreg on the kidney recipient
			phase 1 and phase 2	
*Autologous polyclonal Treg for Lupus*/NCT02428309	NIAID	University of California, San Francisco	July 2015/December 2019/18/phase 1	To evaluate the safety, tolerability, and effect of 3 different doses of *ex vivo*-expanded autologous polyclonal Treg therapy in adults with skin (cutaneous) involvement of their lupus

*NIAID, National Institute of Allergy and infectious Diseases; Treg, regulatory T cell; darTreg, donor alloantigen reactive regulatory T cell*.

Clinical trials of Treg therapy for solid organ transplantation and autoimmune diseases that are listed on www.ClinicalTrials.gov and currently recruiting are summarized in Table 1.

## Summary

Overall, Treg hold promise as personalized therapy in the treatment of liver diseases and, in the future, may be applied in the non-transplant as well as post-transplant setting to overcome or minimize the use of broad spectrum immunosuppressant medications that have unfavorable side effects (79). Their mechanisms of function vary at different tissues. While Treg therapy has been applied in other autoimmune disease settings, such as diabetes and SLE, successful application in the treatment of the liver diseases, including autoimmune-related liver patients, may require consideration of the impact of the inflamed liver microenvironment itself which is enriched with cytokines, microbes, and metabolites. Dissecting the clonotype and gene signature would facilitate exploration of antigen specificity, and therapy can be optimized by administering antigen-specific Treg. Notably, it is already clear that the effective proliferation and function of Treg is dependent on IL-2, yet the liver microenvironment is deficient in IL-2 (15). Thus, it is anticipated that Treg therapy for liver diseases would benefit from adjuvant supply of low-dose IL-2, which can selectively potentiate Treg function without establishing global immune activation. It is now an exciting time to conduct Treg cell therapy with manipulation of different cytokines and the microenvironment to achieve a successful and potential cure in patients with AILD.

## Author Contributions

All authors listed have made substantial, direct, and intellectual contribution to the work and approved it for publication. The views expressed in this paper are those of the author(s) and not necessarily those of the NHS, the NIHR, or the Department of Health.

## Conflict of Interest Statement

The authors declare that the research was conducted in the absence of any commercial or financial relationships that could be construed as a potential conflict of interest.
